# Leaky Vaccines Protect Highly Exposed Recipients at a Lower Rate: Implications for Vaccine Efficacy Estimation and Sieve Analysis

**DOI:** 10.1155/2014/813789

**Published:** 2014-05-07

**Authors:** Paul T. Edlefsen

**Affiliations:** Fred Hutchinson Cancer Research Center, Seattle, WA 91809, USA

## Abstract

“Leaky” vaccines are those for which vaccine-induced protection reduces infection rates on a per-exposure basis, as opposed to “all-or-none” vaccines, which reduce infection rates to zero for some fraction of subjects, independent of the number of exposures. Leaky vaccines therefore protect subjects with fewer exposures at a higher effective rate than subjects with more exposures. This simple observation has serious implications for analysis methodologies that rely on the assumption that the vaccine effect is homogeneous across subjects. We argue and show through examples that this heterogeneous vaccine effect leads to a violation of the proportional hazards assumption, to incomparability of infected cases across treatment groups, and to nonindependence of the distributions of the competing failure processes in a competing risks setting. We discuss implications for vaccine efficacy estimation, correlates of protection analysis, and mark-specific efficacy analysis (also known as sieve analysis).

## 1. Introduction

Public health vaccines have reduced the global burden of disease considerably over the past century. Statistical design and analysis of vaccine efficacy trials are well-studied and critical components of the development of these interventions. As discussed in [1], analysis of vaccine interventions is usually complicated by the unobservability of exposure. Even when exposure rates are constant across subjects, the stochastic nature of exposures means that some subjects will experience no exposures while others may experience multiple exposures. Except in challenge trials in which exposure is controlled by the experimental setting, or in controlled scenarios in which exposure is estimable; the missingness of exposure times poses a challenge to estimation of per-exposure vaccine efficacy.

Vaccine efficacy has multiple definitions (see [2] for a thorough review), including per-exposure reduction in susceptibility, which is distinct from reduction in instantaneous hazard of infection and also from reduction in overall (attack) rate of infection. These definitions coincide in some settings but generally are not the same. It has been shown that the mechanism of the vaccine's protection is relevant to the relationship among these kinds of efficacy, with “leaky” vaccines (defined as those modifying per-exposure infection rates for all subjects equally) at one extreme and “all-or-none” vaccines (which completely protect some subjects and have no effect on the others) at the other extreme. While for all-or-none vaccines the overall attack rate is reduced by the fraction of recipients that have protective responses, for leaky vaccines the attack rate is reduced by an amount that depends on the number of exposures that each subject experiences.

If each subject experiences exactly one exposure during the trial, then a leaky vaccine reducing susceptibility by 50% has the same attack-rate efficacy as an all-or-none vaccine that fully protects 50% of the subjects. Here we focus on examples such as HIV-1 vaccine trials, in which multiple exposures are possible and in which some (or many) participants will experience no exposures at all. In such settings, the effect of a partially efficacious leaky vaccine is to reduce attack rates for subjects who experience one exposure more than for subjects who experience multiple exposures, since each exposure has an independent opportunity to infect. Although in this setting reinfection is possible, we assume that the endpoint of interest is initial infection only, so that infected subjects are removed from the at-risk population.

In this paper, we consider the analysis of leaky vaccines when there is heterogeneity in subjects' infecting exposure distributions (defined as either heterogeneity in exposure or in per-exposure infection susceptibility or both). Through arguments and simulation, [3] have previously shown that, for this scenario, the assumption of proportional hazards (that is usually required for Cox modeling) is violated. Here we restate these arguments and consider additional implications for survival analysis in the setting of competing risks. We argue that the conditional distribution of exposure rates, given infection status, depends on both time and treatment assignment, which implies not only that the hazard ratio varies over time (reflecting variation in the risk group distribution among the “at-risk” uninfected population) but also that the risk group distribution varies among those infected—both over time and across treatment groups. We discuss general implications of this observation for vaccine efficacy analysis methods and for immune correlates analysis methods, including case-only methods (which save resources by evaluating covariates only among the subjects who became infected in a trial), and for competing risks settings. We review proofs for two mark-specific efficacy analysis (also called “sieve analysis”) methodologies and show that the proofs do not apply in this setting, leading to a potential bias in these analyses. We conclude that in the absence of exposure data, failure time and failure type data alone are insufficient to distinguish per-exposure vaccine efficacy that varies across subjects from per-exposure vaccine efficacy that varies across marks of the failure.

## 2. Materials and Methods

### 2.1. Notation and Definitions

In this section, we introduce the notation and examples that we will use to demonstrate the implications of risk heterogeneity for evaluating the efficacy of leaky vaccines. We assume a setting of a well-conducted placebo-controlled randomized clinical trial to evaluate a vaccine intervention, where the effect of the intervention is to reduce the per-exposure infection probability by a (multiplicative) factor *η*, so that if for a subject the probability of infection given one exposure is *ϕ*_*p*_ in the absence of the intervention, it is *ϕ*_*v*_ = *ηϕ*_*p*_ if the subject is assigned to the vaccine treatment group.

As shown in [1], for nonharmful vaccines, the vaccine effect can be seen as a filter on each subject's infecting exposure process *N*, which is itself a filtered version of the exposure counting process *E*. That is, for an arbitrary process *E*(*t*) counting a placebo recipient's exposures up to time *t*, an infection occurs with probability *ϕ*_*p*_ for each time *t* at which the exposure count increases. For vaccine recipients this probability is reduced to *ϕ*_*v*_ = *ϕ*_*p*_ *η*, where with probability 1 − *η* the would-be-infection is avoided due to the vaccine intervention. With minor adjustments the arguments in this paper can be adapted to apply to vaccines that could induce harm, such that the vaccine is not providing an additional filter but is modifying and possibly increasing the rate at which exposures become infections; for simplicity of presentation we will proceed with the assumption that 0 < *η* < 1.

We assume that we do not observe the exposure processes at all; we are given data of the form of per-subject pairs (*T*, *M*) representing the observed part of the latent pair of processes (*I*, *C*), where *I* is the time at which the subject's infection count *N* increases from zero to one and *C* is the right-censoring time. We only observe one value of this latent pair, *T* ≡ min⁡⁡(*I*, *C*). *M* = 1 indicates missingness of the infection time (*M* = 1 means that *T* = *C*). We assume conditions of noninformative censoring, such that *I* ⫫ *C*. The arguments are easily extended to a setting in which the right-censored values are used to improve estimates of efficacy, but henceforth we consider only the uncensored data (except when explicitly addressing the assumption in the context of competing risks analysis).

We define three distinct notions of vaccine efficacy, based on different quantities. First we define the attack rate for treatment group *x* (vaccine recipients have *x* = *v* and placebo recipients have *x* = *p*) as *a*_*x*_ = Pr(*T* < *τ* | *x*). Then the attack-rate vaccine efficacy VE^*a*^ = 1 − *a*_*v*_/*a*_*p*_ is the reduction in the total fraction of infected subjects due to the vaccine. The per-exposure vaccine efficacy VE^*ϕ*^ = 1 − *ϕ*_*v*_/*ϕ*_*p*_ is the reduction in the per-exposure susceptibility to infection due to the vaccine. Finally we define the hazard-rate vaccine efficacy VE^*λ*^ = 1 − *λ*_*v*_(*τ*)/*λ*_*p*_(*τ*), where for each treatment group *x* the infection hazard is *λ*_*x*_(*t*) = lim⁡_*d*↘0_Pr(*N*(*t* + *d*) = 1 | *x*, *N*(*t*) = 0)/*d*, the instantaneous rate of infection just after time *t* given noninfection up to time *t*. The set of subjects with treatment assignment *x* that are not infected up to time *t* is called the at-risk group *R*_*x*_(*t*), and the set of subjects already infected by time *t* is called the infected group *I*_*x*_(*t*).

### 2.2. Risk Groups

We assume for simplicity of presentation that there are two risk groups. We allow that some fraction *π*_*h*_ of subjects is “high risk,” by which we mean that the exposure rates are higher for these subjects, and in particular that both single and multiple exposures are more likely for these subjects. Since a “leaky” vaccine only protects a subject if every exposure is noninfecting, the attack-rate VE is higher for low-risk subjects than for high-risk subjects. For example, if the vaccine effect reduces per-exposure susceptibility by *η* = 50%, and if low-risk subjects tend to have about one exposure during the trial and high-risk subjects tend to have about nine exposures, then about half of the low-risk subjects will be protected while about 0.5^9^ = 0.2% of the high-risk subjects will be protected. This implies that the fraction of high- (versus low-) risk subjects among the infected vaccine recipients will differ from that fraction among the infected placebo recipients.

For illustration, we suppose arbitrarily that the baseline hazard function is constant, as in the exponential model. Under this model, an infection event occurs in a low-risk placebo recipient at a time-constant rate *λ*_*l*_, which can be written as the low-risk marginal rate of an exposure *λ*_*l*_^*E*^ times the conditional probability *ϕ*_*lp*_ that the exposure will infect the low-risk placebo recipient: *λ*_*l*_ ≡ *λ*_*l*_^*E*^ *ϕ*_*lp*_. For high-risk subjects we have corresponding infecting exposure rate *λ*_*h*_ ≡ *λ*_*h*_^*E*^ *ϕ*_*hp*_. Because of the memoryless property of the exponential model, these rates are equivalently viewed as hazards.

The fraction *a*_*lp*_ of low-risk placebo recipients that will become infected is the fraction having infecting exposure times *T* that exceed the trial duration *τ*. Since we assume independence across subjects, under the exponential model the number of infected low-risk placebo recipients follows a binomial distribution with proportion *a*_*lp*_ given by the probability that a Poisson-distributed random variable (with rate *λ*_*l*_ *τ*) counting infecting exposures exceeds zero: *a*_*lp*_ ≡ 1 − *e* ^−*λ*_*l*_*τ*^. Similarly the number of the high-risk placebo recipients that will become infected is given by a binomial distribution with proportion *a*_*hp*_ ≡ 1 − *e* ^−*λ*_*h*_*τ*^.

### 2.3. V*E* ^*a*^ under Heterogeneous Risk

A leaky vaccine with multiplicative vaccine effect *η* = *ϕ*_*lv*_/*ϕ*_*lp*_ = *ϕ*_*hv*_/*ϕ*_*hp*_ (corresponding to a VE^*ϕ*^ of 1 − *η*) will result in overall attack rates *a*_*lv*_ = 1 − *e* ^−*λ*_*l*_*τη*^ and *a*_*hv*_ = 1 − *e* ^−*λ*_*h*_*τη*^ among low- and high-risk vaccine recipients, respectively. If the vaccine is partially efficacious then 0 < *η* < 1, and *a*_*hv*_ < *a*_*hp*_ and *a*_*lv*_ < *a*_*lp*_, so the vaccine reduces the probability of being infected for both high- and low-risk participants. However, the reduction is not the same for high-risk participants as for low-risk participants, since *λ*_*h*_ > *λ*_*l*_ implies that

(1) $$\begin{array}{c} \left(\frac{a_{hv}}{a_{hp}}=\frac{1-e^{-\lambda_{h}\eta \tau }}{1-e^{-\lambda_{h}\tau }}\right)>\left(\frac{a_{lv}}{a_{lp}}=\frac{1-e^{-\lambda_{l}\eta \tau }}{1-e^{-\lambda_{l}\tau }}\right). \end{array}$$

The direction of the inequality is reversed for harmful vaccines (with *η* > 1).

### 2.4. Differential Enrichment of High-Risk Infected Subjects across Treatment Groups

This differential attack-rate efficacy by risk group results in a different proportion of high-risk participants among infected subjects at the end of the trial across the two treatment groups. To see this, consider that if the beginning-of-trial probability of being high risk is *π*_*h*_, then we can define the conditional probability *γ*_*hx*_ of being high risk for subjects in the infected group *I*_*x*_(*τ*) in terms of the posterior odds *γ*_*hp*_/(1 − *γ*_*hp*_) ≡ (*π*_*h*_/(1 − *π*_*h*_))(*a*_*hp*_/*a*_*lp*_) for placebo recipients and *γ*_*hv*_/(1 − *γ*_*hv*_) ≡ (*π*_*h*_/(1 − *π*_*h*_))(*a*_*hv*_/*a*_*lv*_) for vaccinees.

For partially efficacious vaccines with 0 < *τ* < 1, since the vaccine reduces low-risk infections more than high-risk infections, *a*_*lv*_/*a*_*lp*_ < *a*_*hv*_/*a*_*hp*_. This results in an enrichment of high-risk participants among the infected vaccinees as compared with the infected placebo recipients: *γ*_*hv*_ > *γ*_*hp*_. For a harmful vaccine, this inequality is reversed.

### 2.5. Differential Enrichment of High-Risk at-Risk Subjects across Treatment Groups

This correspondingly results in a different proportion of high-risk participants among subjects remaining at-risk at the end of the trial across the two treatment groups. The posterior odds of being high risk among those remaining uninfected are *ω*_*hp*_/(1 − *ω*_*hp*_) ≡ (*π*_*h*_/(1 − *π*_*h*_))((1 − *a*_*hp*_)/(1 − *a*_*lp*_)) for placebo recipients and *ω*_*hv*_/(1 − *ω*_*hv*_) ≡ (*π*_*h*_/(1 − *π*_*h*_))((1 − *a*_*hv*_)/(1 − *a*_*lv*_)) for vaccinees.

If (1 − *a*_*hp*_)/(1 − *a*_*lp*_) < (1 − *a*_*hv*_)/(1 − *a*_*lv*_), or equivalently if (1 − *a*_*lv*_)/(1 − *a*_*lp*_) < (1 − *a*_*hv*_)/(1 − *a*_*hp*_), then this results in an enrichment of high-risk participants among the uninfected vaccinees as compared with the uninfected placebo recipients: *ω*_*hv*_ > *ω*_*hp*_. This condition is met if both *a*_*lv*_/*a*_*lp*_ < *a*_*hv*_/*a*_*hp*_ and (*a*_*hp*_ − *a*_*hv*_)>(*a*_*lp*_ − *a*_*lv*_), since we can write

(2) $$\begin{array}{c} \frac{1-a_{lv}}{1-a_{lp}}<\frac{1-a_{hv}}{1-a_{hp}}\\ \text{as}\;\;a_{lv}a_{hp}+\left(a_{lp}-a_{lv}\right)<a_{hv}a_{lp}+\left(a_{hp}-a_{hv}\right). \end{array}$$

For a partially efficacious vaccine we have shown that *a*_*lv*_/*a*_*lp*_ < *a*_*hv*_/*a*_*hp*_, which implies that *a*_*lv*_ *a*_*hp*_ < *a*_*hv*_ *a*_*lp*_, so if also (*a*_*hp*_ − *a*_*hv*_)>(*a*_*lp*_ − *a*_*lv*_), then the condition in (2) is satisfied.

We may still have *ω*_*hv*_ > *ω*_*hp*_ despite not satisfying (*a*_*hp*_ − *a*_*hv*_)>(*a*_*lp*_ − *a*_*lv*_) and *a*_*lv*_/*a*_*lp*_ < *a*_*hv*_/*a*_*hp*_. The general condition is that

(3) $$\begin{array}{c} a_{hv}a_{lp}-a_{lv}a_{hp}>\left(a_{lp}-a_{lv}\right)-\left(a_{hp}-a_{hv}\right). \end{array}$$

### 2.6. Summary

In this section we have shown that, for leaky vaccines, subject heterogeneity in risk results in time variation of VE^*a*^ = 1 − *a*_*v*_/*a*_*p*_, where the values *a*_*x*_  (for *x* ∈ {*v*, *p*}) are the marginal attack rates for vaccine and placebo recipients. We have shown that this implies a change in the composition of both the infected group *I*_*x*_(*t*) and in the at-risk group *R*_*x*_(*t*) over time such that for both vaccine and placebo recipients the proportion of high-risk subjects is higher in the infected group than in the at-risk group by the end of the trial. We have shown that this effect differs by treatment group such that for partially protective leaky vaccines, the fraction *γ*_*hv*_ of high-risk subjects among those infected in the vaccine group is higher than the fraction *γ*_*hp*_ of high-risk subjects among those infected in the placebo group. Finally we have shown that the proportion of high-risk subjects among those remaining at-risk at the end of the trial may be higher or lower in the vaccine group as compared with the placebo group; the crucial point is that in general one should not expect that the at-risk groups have the same distribution of high-risk subjects across treatments arms.

## 3. Results and Discussion

Next, we turn to implications of these observations. First, we show, as has been shown previously, that VE^*λ*^ changes over time or equivalently that the hazard proportion is inconstant. Then we discuss implications of the risk imbalance in the infected group for introducing bias into correlates of protection analysis whenever a putative correlate of protection is also a correlate of placebo-recipient risk. Finally, we discuss implications of the risk imbalance in the at-risk group in a competing risks analysis and show that this risk imbalance violates conditions required for the correctness of proofs of unbiasedness for two sieve analysis methods for leaky vaccines, with the implication that the proven unbiasedness is only guaranteed if subject risk is homogeneous.

### 3.1. Implications for the Proportional Hazards Assumption

The differential efficacy for high-risk and low-risk subjects has the effect of inducing a violation of the proportional hazards assumption for the marginal hazards, even if it holds separately for the low-risk hazards and the high-risk hazards. Each marginal hazard function is a mixture of the two risk-group hazards, and the mixing proportion changes over time differently for placebo recipients than for vaccine recipients as the at-risk frequencies diverge due to different rates of infection in the two risk groups.

The marginal hazard rate of infection is a mixture over high- and low-risk subjects. At the beginning of the trial the marginal hazard for placebo recipients is *λ*_*p*_(0) ≡ *π*_*h*_ *λ*_*h*_ + (1 − *π*_*h*_)*λ*_*l*_. This changes over the trial, since *λ*_*p*_(*τ*) ≡ *ω*_*hp*_ *λ*_*h*_ + (1 − *ω*_*hp*_)*λ*_*l*_. The change is due to a shifting mixing proportion, and it appears even when there are constant hazards within each risk group.

For vaccine recipients, there is also a change in the marginal hazard over the course of the trial, but the change is different than for placebo recipients. At the beginning of the trial the marginal hazard for vaccine recipients is *λ*_*v*_(0) ≡ *π*_*h*_ *λ*_*h*_ *η* + (1 − *π*_*h*_)*λ*_*l*_ *η*. At the end of the trial, *λ*_*v*_(*τ*) ≡ *ω*_*hv*_ *λ*_*h*_ *η* + (1 − *ω*_*hv*_)*λ*_*l*_ *η*.

If the study enrolls *n* vaccine recipients, *n*∗*π*_*h*_ of whom are high risk, then a *a*_*lv*_ infection rate among low-risk vaccine recipients (and a corresponding *a*_*hv*_ among the high-risk vaccinees) over the course of the trial yields a difference in the ratio of high : low risk at-risk subjects from *π*_*h*_ at the beginning to *ω*_*hv*_ at the end. Since the high-risk hazard rate is *λ*_*h*_/*λ*_*l*_ times the low-risk hazard rate *λ*_*l*_, then the marginal hazard goes from ((1 − *π*_*h*_)*λ*_*l*_ *η*+*π*_*h*_(*λ*_*h*_/*λ*_*l*_)*λ*_*l*_ *η*)=(1 − *π*_*h*_ + *π*_*h*_(*λ*_*h*_/*λ*_*l*_))*λ*_*l*_ *η* to ((1 − *ω*_*hv*_)*λ*_*l*_ *η*+*ω*_*hv*_(*λ*_*h*_/*λ*_*l*_)*λ*_*l*_ *η*)=(1 − *ω*_*hv*_+*ω*_*hv*_(*λ*_*h*_/*λ*_*l*_))*λ*_*l*_ *η*. The vaccine recipient hazard is 1 − (1 − *ω*_*hv*_ + *ω*_*hv*_(*λ*_*h*_/*λ*_*l*_))/(1 − *π*_*h*_ + *π*_*h*_(*λ*_*h*_/*λ*_*l*_)) times 100% lower at the end of the trial than at the beginning. The placebo recipient hazard is correspondingly 1 − (1 − *ω*_*hp*_ + *ω*_*hp*_(*λ*_*h*_/*λ*_*l*_))/(1 − *π*_*h*_ + *π*_*h*_(*λ*_*h*_/*λ*_*l*_)) times 100% lower at the end of the trial than at the beginning.

Unless the end-of-trial rates of high-risk subjects among the uninfected are the same for both treatment groups (i.e., unless *ω*_*hp*_ = *ω*_*hv*_), the hazard ratio (vaccine to placebo) will also differ at the end of the trial. The marginal hazards ratio at the beginning of the trial is *λ*_*v*_(0)/*λ*_*p*_(0) = (*π*_*h*_ *λ*_*h*_ *η* + (1 − *π*_*h*_)*λ*_*l*_ *η*)/(*π*_*h*_ *λ*_*h*_ + (1 − *π*_*h*_)*λ*_*l*_). At the end of the trial it is *λ*_*v*_(*τ*)/*λ*_*p*_(*τ*) = (*ω*_*hv*_ *λ*_*h*_ *η* + (1 − *ω*_*hv*_)*λ*_*l*_ *η*)/(*ω*_*hp*_ *λ*_*h*_ + (1 − *ω*_*hp*_)*λ*_*l*_). These are equal only when *ω*_*hv*_ = *ω*_*hp*_ = *π*_*h*_, and never for leaky vaccines with heterogeneous risk.

We demonstrate the situation with a simple example of a leaky vaccine with about *a*_*lp*_ = 4% of low-risk placebo recipients becoming infected over the unit-time course of the trial (corresponding to a low-risk infecting exposure rate of *λ*_*l*_ = 0.04) and about *a*_*hp*_ = 36% of high-risk placebo recipients becoming infected (corresponding to a high-risk instantaneous infecting exposure rate of *λ*_*h*_ = 0.446). We suppose a leaky vaccine that reduces the infection probability by *η* = 50% per exposure, which corresponds to *a*_*lv*_ = 2% of low-risk vaccinees and *a*_*hv*_ = 20% of high-risk vaccinees becoming infected over the course of the trial. We suppose that, at the start of the trial, *π*_*h*_ = 5% of participants are high risk.

If the study enrolls 100 vaccine recipients, 5 of whom are high risk, then a 2% infection rate among low-risk vaccine recipients (and a corresponding 20% among the high-risk vaccinees) over the course of the trial yields a difference in the mixture of high : low risk hazards from 5 : 95 (*π*_*h*_ = 5%) at the beginning to 4 : 93 (*ω*_*hv*_ = 4.1%). Since in our example the high-risk hazard rate *λ*_*h*_ is about eleven times the low-risk hazard rate *λ*_*l*_, then the marginal vaccine hazard goes from (0.95 × *λ*_*l*_ *η* + 0.05 × 11*λ*_*l*_ *η*) = 1.5*λ*_*l*_ *η* to (0.96 × *λ*_*l*_ *η* + 0.04 × 11*λ*_*l*_ *η*) = 1.4*λ*_*l*_ *η*. In this example, the vaccine recipient marginal hazard is about 5.8% lower at the end of the trial than at the beginning.

If that study also enrolls 100 placebo recipients, 5 of whom are high risk, then a 4% infection rate among low-risk placebo recipients and a corresponding 36% among the high-risk placebos over the course of the trial yields a difference in the mixture of high : low risk hazards from 5 : 95 at the beginning to 3 : 91 (about *ω*_*hp*_ = 3.4%). Then the marginal placebo hazard goes from (0.95 × *λ*_*l*_ + 0.05 × 11*λ*_*l*_) = 1.5*λ*_*l*_ to (0.976 × *λ*_*l*_ + 0.034 × 11*λ*_*l*_) = 1.34*λ*_*l*_. In this example, the marginal placebo recipient hazard is about 10.7% lower at the end of the trial than at the beginning.

For the conditions of our example, the hazard ratio at the beginning of the trial (vaccine/placebo) is 1.5*η*/1.5 = 0.5, but at the end of the trial it is 1.4*η*/1.34 = 0.527, about a 5.5% increase. If we increase the rate of exposures for high-risk subjects *λ*_*h*_ to 1, so that we expect about one exposure per high-risk participant, then the ending hazard ratio is about 18.9% higher than it is at the trial's beginning. The discrepancy peaks at about *λ*_*h*_ = 3, at a 55% increase in the hazard ratio, then decreases again as the leaky vaccine effect diminishes for the high-risk subjects. At *λ*_*h*_ = 10, the ending hazard proportion is down to 8.8% above its starting value.

**Figure 1:**
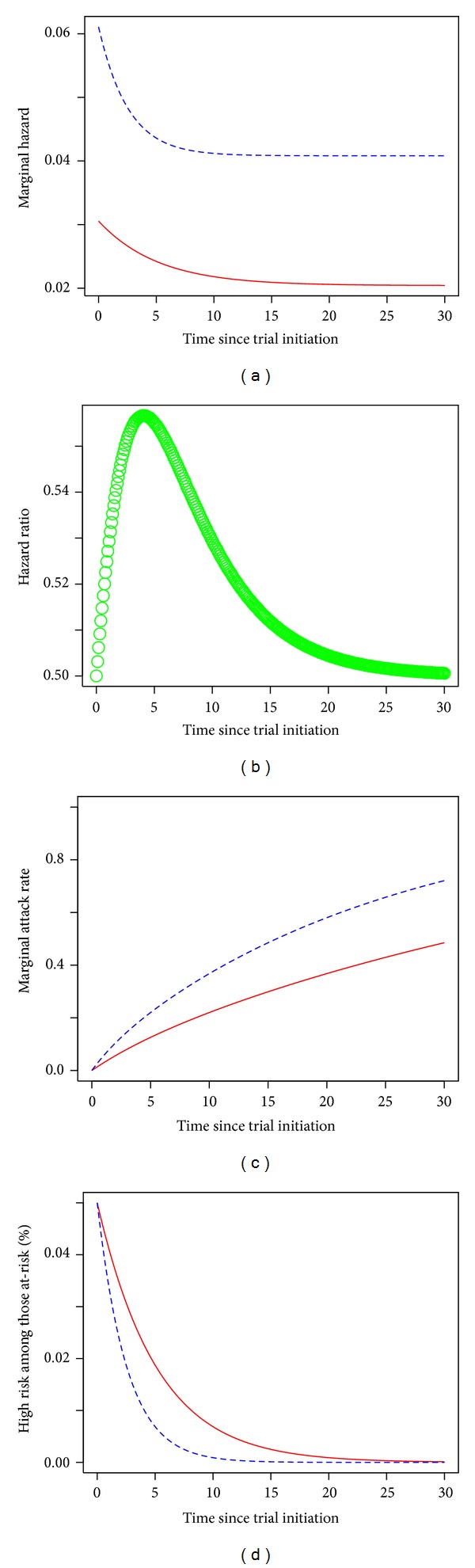
Effects of differential enrichment for high-risk subjects in the at-risk population across treatment groups. (a) The marginal hazards as a function of time for placebo recipients (dashed blue line) and vaccine recipients (solid red line) for the conditions of our example trial in which a 1 : 1 randomization allocates subjects to receive a placebo or a leaky vaccine with per-exposure efficacy *η* = 0.5, with independent Poisson exposure rates *λ*_*l*_ = 0.0408 and *λ*_*h*_ = 0.4463 for low-risk and high-risk subjects, respectively, and a *π*_*h*_ = 5% starting fraction of high-risk subjects. (b) The ratio of the marginal hazards in (a). (c) The fraction of subjects infected in the two groups over time. (d) The proportions *ω*_*hp*_(*t*) and *ω*_*hv*_(*t*) of the at-risk groups *R*_*p*_(*t*) (dashed blue line) and *R*_*v*_(*t*) (solid red line) that are high risk, over time.

The hazard proportion also changes over the duration of the trial; Figure 1 shows the change over time of the hazard ratio. The plot shows that the change is nonmonotonic in time and that it has a single mode and a right skew. For harmful vaccines with *η* > 1, the plot has the same shape, but the plot is mirrored over the *X* axis, with negative percent change values indicating that the hazard ratio decreases and then increases again.

### 3.2. Implications for Correlates Analysis

The differential enrichment of high-risk subjects among those infected across treatment groups implies that even if a vaccine has an equal per-exposure effect on every subject, its effects on overall attack rates are expected to differ by risk group. When evaluating a vaccine candidate to determine if its partial efficacy can be attributed to unequal vaccine effects across subjects (by for instance identifying preexisting subject traits or immune responses to vaccination that differentiate subjects for whom the vaccine worked best), care must be taken to differentiate between these expected attack-rate effects (which do not reflect differential per-exposure efficacy by subject trait) from effects that truly modify the per-exposure efficacy by subject trait.

Several authors have noted that the analysis of vaccine trials to identify subject correlates of VE^*ϕ*^ is complicated by missingness of the counterfactual effects of vaccination on the placebo recipients (see [4] for a review and unifying perspective). With the data typically available from a clinical trial it is possible to estimate correlates of infection risk within vaccine recipients and placebo recipients separately but without strong assumptions or additional data it is not possible to causally attribute changes in infection risk (for some subset of subject covariates) to the vaccine treatment assignment. The problem is that it is not possible to differentiate between preexisting risk differences and vaccine-induced risk differences without additional information.

Here we point out that leaky vaccines with heterogeneous subject risk constitute a concrete example of this difficulty. Since we expect differential enrichment of high-risk subjects even when the vaccine has an equal per-exposure effect, then any correlate of infection risk in the placebo group will necessarily correlate with VE^*a*^. We also expect a correlation between a subject's risk category and VE^*λ*^. The implication is that (in the absence of additional justification) any identified correlate of risk in the vaccine group should not be interpreted as a correlate of protection if it is also a correlate of risk in the placebo group.

This suggests a test for any putative candidate correlate of VE^*ϕ*^: if an association exists between the correlate and infection risk in the placebo group then any correlation observed in the vaccine group (even a much stronger correlation) may be solely attributable to expected risk group enrichment and should not (without further justification) be attributed to differential per-exposure efficacy. If there is no association in the placebo group then the correlate of vaccine-group infection risk remains a plausible candidate as a correlate of VE^*ϕ*^. Further work is required to develop the conditions under which a vaccine-group correlate of infection risk can be attributed to differential efficacy, but this argument suggests general caution whenever a placebo-group correlation cannot be ruled out.

This reasoning also warrants caution about so-called “case-only” methods, which evaluate only the infected cases. Such methods can be cost saving because correlates need not be measured in uninfected subjects. However if there is enrichment of different risk groups among infected subjects in the two treatment groups (which should be expected for any leaky vaccine), then covariate differences across treatment groups among infected subjects may simply reflect differential baseline risk. Since correlate information is unavailable for uninfected subjects, in case-only analyses the test of placebo-recipient risk correlation is not possible. Below we examine a special case of case-only analysis in the setting of competing risks, known as “sieve analysis.”

### 3.3. Implications for Competing Risks and Sieve Analysis

In addition to evaluating vaccine efficacy as a function of subject-specific covariates, it is often of interest to evaluate the extent to which a vaccine's efficacy differs by type of infection. In a series of papers on what has variously been called “mark-specific intervention efficacy” or “sieve effects,” Gilbert et al. defined sufficient conditions under which estimates are unbiased for quantities relevant to the identification of these effects [5–7]. Here we argue that one of those conditions can be represented as a requirement of “proportional exposure pseudohazards” and that this condition is required not only for the failure-type-only methods (such as multinomial logistic regression (MLR)) but also for the time-to-event methods (including competing risks Cox models, even when relaxing the assumption of proportional baseline risks as in [8]). In the special case of a leaky intervention, this is equivalent to a condition that we call “balanced replacement,” which requires that for each subject, the exposure type be independent of the exposure time and exposure history. We show that if there is subject variation in infection risk, then even balanced replacement is insufficient to ensure the proportional pseudohazards condition.

A sieve effect is defined as any violation of equivalence of VE_*s*_^*ϕ*^ across mark types *s* [6]. We define per-exposure mark-specific relative risks:

(4) RRϕ(s)≡Pr(fail with type s ∣ one exposure to type s, vaccine recipient)Pr(fail with type s ∣ one  exposure  to  type s, placebo recipient)=ϕvsϕps,

and let VE_*s*_^*ϕ*^ = 1 − RR^*ϕ*^(*s*).

Thus, a sieve effect is defined as a lack of equivalence across all types *s* ∈ 1,…, *J* of RR^*ϕ*^(*s*). In terms of odds ratios to some baseline type (arbitrarily we use type *s* = 1 here), the null hypothesis of no sieve effect is that for all *s*, OR^*ϕ*^(*s*) = 1, where

(5) ORϕ(s)=RRϕ(s)RRϕ(1)=ϕvs/ϕpsϕv1/ϕp1=ϕvs/ϕv1ϕps/ϕp1.

In Appendix B we revisit the proof that under the condition that was called “Assumption 2” in [6, page 804], which “implies that the strain-specific exposure intensities are proportional, that is, *λ*_*Es*_(*t*) = *θ*_*s*_ *λ*_*E*1_(*t*),” estimates of odds ratios based only on the type distributions of observed infections in treated and untreated subjects of a randomized controlled trial are unbiased for OR^*ϕ*^(*s*). The proof establishes an equivalence between the per-exposure odds ratio OR^*ϕ*^(*s*) and two other odds ratios of interest: the “prospective” (or “attack-rate”) odds ratio, OR^*a*^(*s*) and the “retrospective” odds ratio OR^*r*^(*s*), as defined below. We show in Appendix A that the proof not only relies on the assumption, following [9], that for any subject the type-specific exposure hazard is that of a history-independent (zero-order) process, but also that it relies on the stronger assumption that the “exposure pseudohazards” are proportional. Whereas exposure hazards condition on the exposure processes, the pseudohazards condition on the subject's infection count being *N*(*t*) = 0, which depends both on the exposure processes *E* and on the chance of each exposure resulting in an infection.

In Appendix C we show that unless each subject can experience at most one exposure during the trial (a condition that we call “thoroughly rare events”), proportional pseudohazards require independence between each subject's exposure type distribution and the timing of his exposures (a condition that we call “balanced replacement”). As noted in [8], this in turn implies independence between each subject's infection time, *T*, and the mark of his infection, *S*, a condition that could only hold under a null hypothesis of no sieve effects. We then show that the proof requires subject homogeneity in risk. Risk inhomogeneity leads to a violation of the proportional pseudohazards condition, and of *T* ⫫ *S*, even when the balanced replacement condition holds for each risk group (or individual subject) separately.

In Appendix D we revisit the argument that time-to-event methods such as competing risks Cox proportional hazards models can yield unbiased estimates of these quantities even when “Assumption 2” is violated. We argue that the assertion of unbiasedness requires an assumption of “noninformative censoring” when treating infections with some marks as censoring events while evaluating other mark types of infections. Since this implies that *T* ⫫ *S*, we argue that the time-to-event methods are also biased unless Assumption 2 holds. We show that heterogeneity of the intervention effect across subjects will generally lead to a violation of the noninformative censoring assumption.

From these arguments we conclude that existing methods for evaluating hypotheses of sieve effects of leaky vaccines are expected to be biased if there is any subject heterogeneity in risk (or in response to the treatment), or if replacement failures are imbalanced. Gilbert has evaluated bias under violations of Assumption 2 in simulation studies [7, 8] and showed that under the conditions of those simulations the bias is limited to a few percentage points unless the marginal attack rate *a*_*p*_ is substantial, even when some subjects have no response to the intervention at all. However, those simulations ensured equal exposure distributions across subjects and did not carefully control replacement distribution balance. Future work is required to update the simulations to more specifically address issues of balanced replacement and of heterogeneous infecting exposure rates.

Particular caution is warranted when using case-only sieve analysis methods as introduced in [10], to which these arguments doubly apply, since in addition to the cautions expressed about the effects of risk group enrichment on case-only methods, the proof of the method's approximate unbiasedness depends on the assumption that individual mark-specific hazards can be evaluated by censoring other marks, using the noninformative censoring assumption. Since that assumption is surely violated whenever there are sieve effects, the use of the case-only sieve analysis method to evaluate sieve effects for subject-genotype dependency as the authors propose (or any other correlate) is not justified by the proof. Even under the null hypothesis of no sieve effects, the arguments presented here and in Appendix D show that the noninformative censoring assumption would only be reasonable in a setting in which types of distributions do not vary by risk group. If the different risk groups tend to be infected by different distributions of viruses even in the absence of treatment, as may be the case for HIV-1 trials (where risk is associated with mode of transmission, which in turn is associated with different populations of viruses), the assumption is likely violated.

It remains likely that these methods, though not proven unbiased, retain their power to detect sieve effects under the heterogeneous risk conditions that we have considered. Although the conditions of those proofs may not hold under heterogeneity, we have not proven the contrary assertion; other proofs that establish conditions under which unbiased estimation is robust to subject variation in risk may yet be devised. Also, in practice absolute unbiasedness may not be required; with further work evaluating the practical implications of these insights, we expect that these methods will be approximately unbiased for many or most applications to leaky vaccines with heterogeneous risk. It remains to future work to conduct a thorough evaluation of the loss of power or the potential anticonservatism of analyses that assume risk homogeneity when the assumption is not justified.

## 4. Conclusions

In this paper we have restated the argument that when conducting statistical analysis of vaccine efficacy trials with heterogeneous exposure or susceptibility risk, care should be taken to account for the putative mechanism of the vaccine. Two extremes of the spectrum of vaccine mechanisms are considered. At one extreme (all-or-none), a vaccine protects some fraction of subjects completely and the remaining fraction are unaffected by it. At the opposite extreme (leaky), a vaccine reduces the per-exposure transmission rate for all recipients equally. We have shown that leaky vaccines induce a violation of the proportional hazards condition that is often assumed in survival analysis, due to a changing fraction of at-risk subjects over time in both vaccinated and unvaccinated individuals. Since these fractions change over time differently in the two treatment groups, even if the proportional hazards condition holds for each risk group individually, the marginal hazard ratio changes over time.

Another effect of subject risk heterogeneity in leaky vaccine trials is that the relative proportions of the risk groups among infected subjects changes over time. We showed that associations between subject covariates and vaccine efficacy will be biased unless those covariates are distributed equivalently in all risk groups. A simple diagnostic analysis of the risk of infection among placebo recipients as a function of the covariate could be used to reject the hypothesis of independence that is required for interpreting correlations with vaccine efficacy as indicative of differential efficacy rather than differential baseline risk, but this is not possible in a “case-only” analysis (which evaluates the association only among infected subjects). This argument cautions against case-only analysis of correlates of the partial efficacy of leaky vaccines when there is subject heterogeneity in risk.

We also addressed the context of competing risks and showed that leaky vaccines with risk heterogeneity will induce time variation in the relative proportion of marks (types of the competing risks) of infections and that since this time variation occurs at different rates in the vaccine and placebo groups, this induces a violation of the equivalence between observable relative attack rates and unobservable per-exposure relative risks that is required for unbiased analysis of mark-specific vaccine efficacies (called “sieve effects” when they differ across types) [6]. Furthermore, this scenario has implications for the commonly encountered analysis methodology of analyzing one mark type of the competing risks by treating the infections by any other type as right-censoring events. In particular, the censorship process will not be independent of the infection process unless the infection times of the competing risks are independent, but the changing fractions of risk groups among the at-risk subjects induce dependence (even when the processes are conditionally independent).

Longini and Halloran [11] introduced an approach (frailty models) to evaluating a vaccine's efficacy when subject susceptibilities in any treatment group vary (with some fraction experiencing complete immunity as in an all-or-none vaccine and the remainder having some per-exposure susceptibility that may vary across individuals and differently for each treatment group). This approach enables estimation of more complex vaccine effects, but the observations in this paper about the implications of leaky vaccines with subject heterogeneity in risk apply to these mixture models too, so for instance a relative enrichment of high-risk subjects among infected vaccine recipients cautions against naive correlation of risk-dependent covariates with infection outcomes.

Recent work has introduced sieve analysis methods for nonleaky vaccines, which have all-or-none style protection but perhaps against only a subset of risk mark categories (in which case they are called “some-or-none” vaccines) [12]. The all-or-none and some-or-none scenarios also engender differential enrichment of high-risk subjects among infected (and also at-risk) subjects, but because any protected subject is fully protected against the vaccine-targeted mark types, the attack rate will be reduced equally across risk groups as long as risk is independent of relative exposure rates. If, however, risk (in terms of rates of infection) is associated with the mark among placebo recipients, as expected for example, in HIV-1 vaccine trials, then the vaccine will reduce infection rates for one risk group more than another.

The arguments in this paper together imply that it is generally not possible to differentiate between mark-specific efficacy and subject-covariate-specific efficacy using failure time and failure type data alone unless subject risk is homogeneous. The only exception is when risk groups (though heterogeneous in overall failure rate) have homogeneous relative rates of the marks of infecting exposures across competing risk mark types. Future work is needed to develop statistical analysis methods that account for both subject heterogeneity (as in a frailty model) and competing risks such that the effects of each can be differentiated in an analysis of a partially efficacious vaccine. Such approaches would likely require parameterization not just of the frailty model but also of the exposure processes, requiring considerable modeling effort and sensitivity analysis.

## Appendices

### A. Correction to the Definition of Proportional Exposure Pseudohazards

In practice we are usually unable to observe exposure events (as noted in [9]), complicating estimation of the per-exposure probabilities of failure *ϕ*_*xs*_. We observe the “retrospective” mark type distributions *P*_*xs*_^*r*^ among those who become infected before the end of trial:

(A.1) Pxsr≡Pr(infected  with  mark s ∣ infected in [0,τ],  treatment  assignment  is x).

This is distinct from the “prospective” (or “joint attack rate”) mark type distribution *P*_*xs*_^*a*^, which is the joint probability of infection (“failure”) (occurring at all) and that the failure is of type *s*. It can also be defined in terms of the “retrospective” failure type distribution *P*_*xs*_^*r*^ and the marginal failure probability *a*_*x*_, since

(A.2) ax≡Pr(failed  in[0,τ] ∣ x),Pxsa≡Pr(fail with type s  in[0,τ] ∣ x)=ax×Pasr.

Both the retrospective and prospective probabilities are distinct from the per-exposure probabilities that we intend to estimate. Gilbert et al. showed in [6] that, under certain conditions, there is an equivalence between the odds ratios for all three of these. The proof (repeated below in Appendix B) begins with an expression of the failure hazard as a function of the type-specific rate of exposure *λ*_*Es*_(*t*) and the per-exposure probabilities of failure *ϕ*_*xs*_ [6, page 805]. With *T* defined as the time of the subject's first failure, *S* the mark of that failure, and *τ* defined as the duration of the clinical trial, he wrote that, for any *t* ∈ [0, *τ*],

(A.3) Pr(t≤T<t+Δt,S=s ∣ T≥t,x)  =Pr(exposed  to  type s in [t,t+Δt) ∣ x,     exposure  history)   ×Pr⁡(t≤T<t+Δt,S=s ∣ T≥t,x,  exposed  to  s  in  [t,t+Δt),  exposure  history).

We note that the first term on the right hand side should include the condition *T* ≥ *t* (that the subject has not yet experienced a failure as of time *t*). This is not subsumed by “exposure history” because it depends not only on the exposure process(es) but also on the per-exposure probabilities of failure. Also, the “exposure history” condition does not exist on the left-hand side of the equation, so it should either be added there or removed from the right-hand side. That is, we can define the exposure-history conditional failure hazard as

(A.4) Pr⁡(t≤T<t+Δt,S=s ∣ T≥t,x,exposure  history)  =Pr(exposed  to  type  s  in  [t,t+Δt) ∣ T≥t,x,      exposure  history)   ×Pr⁡(t≤T<t+Δt,X=s ∣ T≥t,x,      1in  exposed  to s in  [t,t+Δt),      exposure  history),

and then define the* marginal* failure hazard *λ*_*xs*_(*t*) in terms of this as

(A.5) ∑exposure historyPr(t≤T<t+Δt,S=s ∣ T≥t,x,      exposure  history)   ×Pr(exposure history ∣ T≥t,x).

Since the per-exposure failure probability is assumed to be independent of exposure history, we can directly define the marginal failure hazard by dropping the condition from the right-hand side:

(A.6) λxs(t)≡Pr(t≤T<t+Δt,S=s ∣ T≥t,x)=Pr(exposed  to  type s in [t,t+Δt) ∣ T≥t,x) ×Pr(t≤T<t+Δt,S=s ∣ T≥t,x,    exposed  to s in [t,t+Δt)).

We define the first term of this corrected failure hazard as the “exposure pseudohazard”:

(A.7) $$\begin{array}{c} l_{xs}\left(t\right)=\text{Pr}\left(\text{exposed}\;\text{to}\;\text{type}\;s\;\text{in}\;\left[t,t+\Delta t\right)\;|\;T\geq t,x\right). \end{array}$$

This is distinct from the generalized hazard function of the type *s* exposure process *E*_*s*_:

(A.8) $$\begin{array}{c} \text{Pr}\left(\text{exposed}\;\text{to}\;\text{type}\;s\;\text{in}\;\left[t,t+\Delta t\right)\;|\;x,\text{exposure}\;\text{history}\right), \end{array}$$

which conditions only on its own history (a generalization of the standard hazard function's dependence on nonfailure to time *t*). There is no difference if every exposure results in a failure, but the two functions depart whenever any exposure event could be avoided (by a roll of the “leaky” dice, with probability 1 − Pr(*t* ≤ *T* < *t* + Δ*t*, *S* = *s* | *T* ≥ *t*, *x*, exposed to *s* in [*t*, *t* + Δ*t*)) = 1 − *ϕ*_*xs*_).

Conceptually, if exposures are occurring that do not result in failures (we call these “avoided failures”), then the subject may nevertheless fail, but later than she would have otherwise. Since lower per-exposure failure probabilities result in more avoided failures, the time-to-event distribution among those who fail in the treated group will be right-shifted compared to what it would have been in the untreated group. We note that it may not be right-shifted for a particular failure type, but aggregating over all types, lower per-exposure failure rates will result in later expected failure times. Mathematically, this dependence between the probability of nonfailure by time *t* (i.e., that *T* ≥ *t*) and the rates of failure avoidance (1 − *ϕ*_*xs*_) can be shown by expanding *l*_*xs*_(*t*) in the equation *λ*_*xs*_(*t*) = *l*_*xs*_(*t*)*ϕ*_*xs*_:

(A.9) lxs(t)=∑exposure historyPr(exposed  to s in       [t,t+Δt) ∣ T≥t,x,         exposure  history) ×Pr(exposure  history ∣ T≥t,x).

By Bayes' theorem,

(A.10) Pr(exposure  history ∣ T≥t,x)  ∝Pr(T≥t ∣ exposure  history,x)   ×Pr(exposure  history ∣ x).

These are not all equal, since if the exposure history includes *k* exposure times *e*_1_,…, *e*_*k*_, then Pr(*T* ≥ *t* | exposure  history, *x*) involves a product of the *k* chances that those failures were avoided and would be monotonically decreasing as the number of exposures increases (except in the boring case of a perfect intervention).

In Appendix B we repeat the proof from [6, 3.12] of the equivalence of the odds ratios, using these corrected definitions. The proof crucially depends on the assumption that the exposure pseudohazards *l*_*xs*_(*t*) are proportional across types. That is, it requires that there exist *J* constants *θ*_*s*_ such that ∀*s*, *l*_*xs*_(*t*) = *θ*_*s*_ *l*_*x*1_(*t*). By (A.9), this would necessitate setting Pr(exposed to *s* in [*t*, *t* + Δ*t*) | *T* ≥ *t*, *x*, exposure history) to depend on exposure history in such a way that exactly counteracts the effect of variation in Pr(exposure  history | *T* ≥ *t*, *x*). This is the condition that we call “balanced replacement” (so-called because it requires that the conditional distribution of failure types to be the same regardless of the number of avoided failures, so “replacement failures” have the same distribution as the failures that they replace through failure avoidance and subsequent reexposure). It is difficult to imagine how this perfect balance could be accomplished other than by assuming complete independence between the type of the exposure and both its timing and the history of the exposure processes (as in independent Poisson-distributed exposure processes for each mark). Effectively, therefore, balanced replacement implies that for each subject (given his treatment and in general his response to the treatment, as discussed below), the time and type of his failures are independent.

### B. Proof of the Equivalence of Odds Ratios under Proportional Pseudohazards

Here we repeat the proof, given in [6], of the equivalence of the retrospective odds ratios and the per-exposure odds ratios. The proof begins by using the equation Pr(*T* ≥ *t* | *x*) = *e* ^−Λ(*t*∣*x*)^ relating a survivor function to a cumulative hazard function to establish that, under conditions of proportional exposure pseudohazards,

(B.1) Pr(T≥t ∣ x)=exp⁡⁡(−∫0tλ(u ∣ x)du)=exp⁡⁡(−∫0t∑lλ(u,l ∣ x)du)=exp⁡⁡(−∫0t∑llxl(u)ϕxldu).

Then the prospective probabilities can be written in terms of the exposure pseudohazards: as

(B.2) Pxsa=∫0τlim⁡Δt↘0⁡Pr(t≤T<t+Δt,S=s ∣ T≥t,x)Δtdt‍=∫0τλ(t,s ∣ x)×Pr(T≥t ∣ x)dt=∫0τlxs(t)ϕxs  Pr(T≥t ∣ x)dt=∫0τlxs(t)ϕxsexp⁡⁡(−∫0t∑llxl(u)ϕxldu)dt.

Then if we define the integrated type *s* exposure pseudohazard *F*_*xs*_(*t*) ≡ ∫_0_^*t*^ *l*_*xs*_(*u*)*du*, we get

(B.3) Pxsa=∫0τlxs(t)ϕxsexp⁡⁡(−∫0t∑llxl(u)ϕxldu)dt=θsϕxs∫0τlx1(t)exp⁡⁡(−∫0tlx1(u)du∑lθlϕxl)dt=θsϕxs∫0τlx1(t)exp⁡⁡(−Fx1(t)∑lθlϕxl)dt=|−1∑lθlϕxlexp⁡⁡(−u∑lθlϕxl)|0Fx1(τ)×θsϕxs=(1−exp⁡⁡(−Fx1(τ)∑lθlϕxl))θsϕxs∑lθlϕxl=(1−Pr⁡(T≥τ ∣ x))θsϕxs∑lθlϕxl=axθsϕxs∑lθlϕxl,

which, since *P*_*xs*_^*a*^ = *a*_*x*_ × *P*_*xs*_^*r*^, implies that

(B.4) $$\begin{array}{c} P_{xs}^{r}=\frac{\theta_{s}\phi_{xs}}{\sum_{l}\theta_{l}\phi_{xl}}. \end{array}$$

This proof relies on the proportional pseudohazards condition to enable the factorization that separates the integrated exposure pseudohazard *F*_*x*1_(*τ*) for an arbitrary mark (*s* = 1) from the time-constant multiples *θ*_*s*_ for *s* > 1 such that *F*_*xs*_(*τ*) = *θ*_*s*_ *F*_*x*1_(*τ*).

Finally, this result guarantees equivalence of retrospective, prospective, and per-exposure odds ratios, since

(B.5) ORr(s)≡Pvsr/PpsrPv1r/Pp1r=Pvsr/Pv1rPpsr/Pp1r=θsϕvs/θ1ϕv1θsϕps/θ1ϕp1=ϕvs/ϕv1ϕps/ϕp1=ORϕ(s), QED.

### C. Conditions for Proportional Pseudohazards

The result of Appendix B is limited to conditions of proportional exposure pseudohazards, which requires balanced replacement. Technically, balanced replacement means that for each subject, given her response to the intervention, any variation in her probability of exposure to a potential failure of type *s* due to dependence on time or exposure history must exactly counterbalance the effects of unavoidable variation in the probability of exposure history over time (conditioned on survival up to that time), such that the relative rate of exposure to one type over another type remains constant. This is accomplished if we assume that the type of the exposure is completely independent of the failure time (and history), an assumption that has been discussed elsewhere [7, 13].

#### C.1. Thoroughly Rare Events

Proportional exposure pseudohazards could also be accomplished if we assume that each subject experiences at most one failure during [0, *τ*] (because in that case, every exposure is a “first exposure” and there are no replacement failures; put another way, in that case the probability of survival given exposure history is effectively independent of exposure history, so there is nothing to balance). It may be tempting to argue that in rare-event settings, this is a reasonable assumption. We note however that the requirement of no replacement failures is stronger than a typical “rare event” scenario, in which the rates *a*_*x*_ are small. The condition we require (thoroughly rare events) means that for all subjects, the probability of a second exposure is zero. While in very-rare event settings, violations of this condition may not lead to bias, it should be noted that the relevant determinant is not the rareness of the event in the total population but the rareness of the event in the subset of subjects who experience a failure during the trial. Since in general, subjects who experience more than one exposure have more chances of failure, in expectation, the probability of experiencing more than one exposure is greater among the subjects who fail than among the larger population. In other words, there is an enrichment of multiply exposed subjects among the subjects who experience a failure during the trial. This is restating our main finding that high-risk subjects are enriched among infected vaccinees.

#### C.2. Risk Homogeneity

Gilbert noted that conditions of the proof require a “leaky” vaccine in which all subjects experience the same intervention effect, and he explored violations of this assumption through simulations [8]. He showed that if some subjects do not have “take” of the intervention (i.e., if there is a chance that an intervention-receiving subject might nevertheless have no change in her per-exposure probabilities of infection), a bias is introduced. Here we show why the proof breaks down in the context of incomplete take. It is analogous to the setting of dichotomous risk, since for high-risk subjects the leaky vaccine's effect on attack-rate vaccine efficacy is reduced, and in some cases it becomes effectively nil.

For the example with two risk groups, the exposure pseudohazard for vaccine recipients becomes

(C.1) $$\begin{array}{c} l_{vs}\left(t\right)=\omega_{hv}\left(t\right)l_{vhs}\left(t\right)+\left(1-\omega_{hv}\left(t\right)\right)l_{vls}\left(t\right), \end{array}$$

where *l*_*vhs*_ and *l*_*vls*_ are the type *s* exposure pseudohazards for high-risk and low-risk subjects, respectively, and

(C.2) ωhx(t) ≡Pr(high  risk ∣ infected  with  mark s by  time  t,x).

For placebo recipients analogously

(C.3) $$\begin{array}{c} l_{ps}\left(t\right)=\omega_{hp}\left(t\right)l_{phs}\left(t\right)+\left(1-\omega_{hp}\left(t\right)\right)l_{pls}\left(t\right). \end{array}$$

Since *ω*_*hp*_(*t*) varies over time and differently from *ω*_*hv*_(*t*), the exposure pseudohazards depend on time and risk group and do not satisfy the proportionality condition, even if the risk group specific-exposure pseudohazards do satisfy it.

### D. Homogeneous Intervention Effects and Proportional Pseudohazards Are Both Required for Noninformative Censoring

Gilbert argued in [8] that some time-to-event methods are robust to violations of the condition that we call “proportional exposure pseudohazards.” He argued that estimates (of retrospective odds ratios) using Cox proportional hazards models without proportional baseline risks could be used to estimate each per-exposure odds ratio in a separate model. So for instance, the per-exposure odds ratio for strain *s* versus strain 1 could be estimated by treating all other types of failure as censoring events. Since the methodology for this estimation requires an assumption of noninformative censoring, this argument breaks down unless the time-to-event distribution of type 1 and type *s* failures is independent of that of the other failure types. Since the condition must hold for all choices of *s*, it effectively requires independence between the time-to-event distribution *T* and the failure type distribution *S*. In the case of a leaky vaccine with homogeneous risk, this can be achieved under the conditions of “balanced replacement” (which requires that the exposure processes exhibit the same sort of time/type independence that is desired for the failure hazard).

We now show that any amount of subject heterogeneity in the intervention effect will lead to a violation of the noninformative censoring assumption, except under the null hypothesis of no sieve effect. We have shown that infection time *T* is not independent from risk group *R*, and so if mark type *S* is also nonindependent from risk, then *S* and *T* will not be independent. If time and type are not independent then if you treat some marks of infection as censoring events, then those events are not independent of the uncensored infection times, and noninformative censoring does not hold.

So it remains to show that failure mark type *S* is not independent of risk. It is clearly the case that if high-risk subjects have a different mark distribution of exposures, then by assumption *S* depends on risk. Also, by the same mechanism of exposure-rate-dependent attack-rate efficacy that is discussed in Section 2, even if high-risk placebo recipients have the same mark distribution of exposures as low-risk placebo recipients, the mark-specific attack-rate vaccine efficacy will vary across the types unless the infecting exposure rates are identical across all types. If the mark distribution of exposures is same for both risk groups and the rate of exposures is constant across marks within each risk group, then only the overall rate of infection varies across risk groups, and the VE_*s*_^*a*^ is the same for all marks. This condition clearly precludes sieve effects.

The noninformative censoring assumption will never hold if there are sieve effects, but even under the null hypothesis of no sieve effects the assumption will only hold in the extreme case in which the (placebo-recipient) infecting exposure processes for all marks are equidistributed within each risk group. As long as some mark exposures occur at higher rates than others, then the attack-rate vaccine effect will differ against the different types, leading to a violation of the noninformative censoring condition.
